# Serial dependencies between form orientation and motion direction are asymmetric

**DOI:** 10.3389/fpsyg.2023.1248307

**Published:** 2023-09-07

**Authors:** Fan-Huan You, Xiu-Mei Gong, Qi Sun

**Affiliations:** ^1^School of Psychology, Zhejiang Normal University, Jinhua, China; ^2^Key Laboratory of Intelligent Education Technology and Application of Zhejiang Province, Zhejiang Normal University, Jinhua, China

**Keywords:** motion direction, form orientation, serial dependence, stimulus reliability, Bayesian inference theory

## Abstract

Much work has been done to uncover the mechanisms underlying form and motion information integration. However, no study examined the symmetry of the integration of form and motion across the temporal domain (i.e., serial dependence). In Experiment 1, we presented form and motion displays sequentially. In the form displays, dot pairs were oriented toward one screen position, indicating the form orientation; in the motion displays, dots moved radially outward. Their motion trajectories were oriented toward one screen position, indicating the motion direction. In each trial, participants reported their perceived form orientation after the form display or their perceived motion direction after the motion display. We found that the current trial’s perceived motion direction was biased toward the previous trial’s form orientation and vice versa, indicating serial dependencies between form orientation and motion direction. In Experiment 2, we changed the form and motion displays’ reliability by varying the two displays’ dot densities. The results showed that the serial dependence of form orientation on motion direction perception decreased only with increasing the current motion display’s reliability; neither the reliability of the previous motion display nor that of the current form display significantly affected the serial dependence of motion direction on form orientation perception. Hence, serial dependencies between form orientation and motion direction were asymmetric. Our across-temporal integrations between form and motion, together with the simultaneous integration of form and motion revealed in the previous studies, depict a comprehensive mechanism underlying the integration of the two pieces of information.

## Introduction

“*A shooting star slipped across the sky with its long tail.*” When we look up at the stars, this beautiful image occasionally projects into our eyes. Our visual system immediately reorganizes the captured visual information: some are organized into the static, e.g., the long tail (motion streak, i.e., form feature); others are moving, e.g., the light moving star (motion feature). Early, it has been proposed that our neural system evolved ventral (“what”) and dorsal (“where”) pathways to process the form and motion features, respectively (Ungerleider and Mishkin, 1982; Mishkin et al., 1983; DeYoe and Van Essen, 1988), supported by many brain-damage cases (Goodale and Milner, 1992; Milner and Goodale, 2006). However, many researchers doubted this proposal and claimed that the processing of the two types of features was closely linked. For example, Or et al. (2010) found that the perception of form orientation was biased toward the concurrently presented motion directions and vice versa (see also Pavan et al., 2017a). Additionally, magnetoencephalography (MEG), transcranial direct current stimulation (tDCS), repetitive transcranial magnetic stimulation (rTMS), and functional magnetic resonance imaging (fMRI) studies have shown that cortical areas V1/V2 and V3b/KO (e.g., Pavan et al., 2017b; Kuai et al., 2020), MT+ (Matsuyoshi et al., 2007; Tang et al., 2015), IPS (Liu et al., 2017) are involved in the integration of the form and motion processing.

Now that many studies have suggested that the processing of form and motion features are inextricably linked, the question arises as to whether the size of the effects of form features on motion feature processing is equal to the size of the effects of motion features on form feature processing. To address this question, Or et al. (2010) showed participants a series of Glass patterns (Glass, 1969) in which all dots were paired orienting toward one orientation, generating a form orientation feature. Meanwhile, all dots were simultaneously moved in one direction, generating a motion direction feature. The form orientation and motion direction were parallel or deflected by several degrees. They asked participants to estimate the form orientation or motion direction. Their results showed that the perceived motion direction was biased toward the form orientation, showing an attractive effect of the form orientation on the motion direction perception (see also Krekelberg et al., 2003; Niehorster et al., 2010; Pavan et al., 2017a; Kuai et al., 2020). In contrast, the attractive effect of the motion direction on the form orientation perception was smaller than the attractive effect of the form orientation on the motion direction perception; or even the perceived form direction was biased away from the motion direction, showing a repulsive effect of the motion direction on the form orientation perception (see also Pavan et al., 2017a). Therefore, Or et al. (2010) found that the mutual integrations between the form orientation and the motion direction were asymmetric.

**Figure 1:**
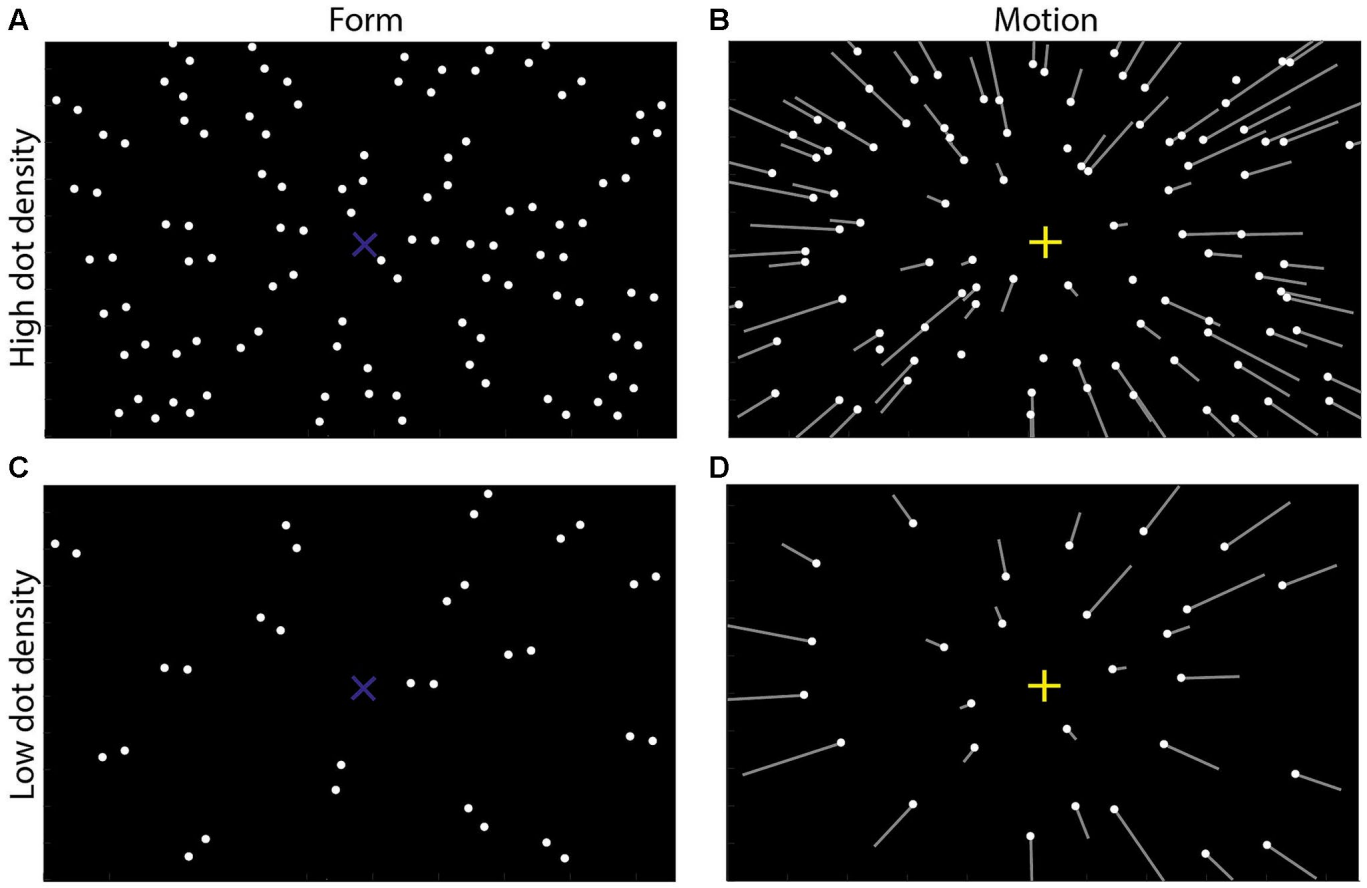
Schematic illustrations of form and motion displays used in Experiment 1. **(A,C)** Form display. Forty-five or 14 dot pairs were included and oriented to one screen position called the form focus of expansion (FoE in short, blue “X,” invisible in the experiment), indicating the form orientation. **(B,D)** Motion display simulated observers translating in a 3D dot-cloud (consisting of 90 or 28 dots) at 1.5 m/s. The white dots indicated the dots’ initial positions at the 1st frame of each display; the white lines illustrated the dots’ motion trajectory in the following frame which were invisible in the experiment. The lengths of the dots indicated the dots’ velocities. All line oriented toward one screen position called the motion FoE (yellow “+,” invisible in the experiment), indicating the motion direction. Note that for clearly showing the differences between the high and low dot-density displays, the dots number in the picture could be a little different from the actual stimuli used in the experiment.

Additionally, some researchers proposed that the integration of the form orientation and the motion direction was consistent with the Bayesian inference theory (Niehorster et al., 2010; Kuai et al., 2020). That is, when one feature (e.g., form orientation) becomes unreliable, participants will rely more on another feature (e.g., motion direction) to make judgments (Cox, 1946; Jaynes, 1986; Bernardo and Smith, 1994; MacKay, 2003). For example, Niehorster et al. (2010) presented participants with Glass patterns, in which all dot pairs were oriented toward one screen position, called the focus of expansion in form display (form FoE in short, blue “×” in Figure 1A). At the same time, all dots moved toward observers in a 3D space, generating a motion pattern that looked like all dots moving radially outward from one screen position, called the focus of expansion in motion display (motion FoE in short, yellow “+” in Figure 1B). They collected participants’ perceived position of the motion FoE and found that they were biased toward the position of the form FoE. Moreover, by making some dots randomly move, they changed the reliability of the motion features. The results showed that the perceived motion FoE was more biased toward the form FoE when the reliability of motion features decreased, suggesting that the integration of motion and form features was consistent with a Bayesian inference account (see also Kuai et al., 2020).

Inspired by the Bayesian inference proposal about the integration of the form orientation and the motion direction, we argued that the asymmetric integrations between the form orientation and the motion direction could be due to the unequal reliabilities of the two features (Or et al., 2010; Pavan et al., 2017a). Although the form and motion features in these studies contain the same number of dots, this cannot guarantee that the reliabilities of the two features were equal. Hence, the effect of the motion direction on the form orientation perception differed from that of the form orientation on the motion direction perception. Therefore, a new method should be developed to examine the symmetry of the mutual integrations between the form orientation and the motion direction.

In addition, researchers presented form and motion displays simultaneously in the above studies. That is, these studies revealed the mechanisms underlying the instant integration of the form orientation and the motion direction. Recently, Wang et al. (2022) first examined how the previously seen form orientation affected the current motion direction perception, which implied the across-temporal integration between form and motion features. On each trial, a form display (Figure 1A) and a motion display (Figure 1B) were presented sequentially (form-load condition), or only a motion display was presented (baseline condition). After the motion display, participants were asked to report their perceived motion direction. Their results showed that, compared with the perceived motion direction in the baseline condition, the perceived motion direction was significantly biased toward the previously presented form orientation, showing an attractive serial dependence of the form orientation on the motion direction perception (see Kiyonaga et al., 2017 for other features). However, they did not examine how the previously seen motion direction affected the form orientation perception, i.e., the serial dependence of the motion direction on the form orientation perception. If there were serial dependencies between the form orientation and the motion direction, whether the sizes of serial dependencies between form and motion perception were equal.

Moreover, similar to the simultaneous integration of form orientation and motion direction, Cicchini et al. (2018) first found that the serial dependence in the orientation perception was also consistent with the Bayesian inference theory. It is known that the discriminations of cardinal orientations are more sensitive than that of oblique orientations (Caelli et al., 1983; De Gardelle et al., 2010), indicating that the cardinal orientations are more reliable (or certain) than oblique orientations. Hence, if serial dependence accords with the Bayesian hypothesis, serial dependence of cardinal orientation would be stronger than that of oblique orientations. Cicchini’s results supported this deduction and were well predicted by a Bayesian ideal observer model, which were reproduced in other studies with other features (van Bergen and Jehee, 2019; Xu et al., 2022).

However, the effectiveness of the Bayesian inference account for serial dependence has been questioned. One reason is that some studies have found that serial dependence is primarily affected by the reliability of the features of the current trials. Varying the reliability of the features of the previous trials does not change the size of serial dependence (Ceylan et al., 2021; Gallagher and Benton, 2022). This contradicts the bidirectional idea of the Bayesian inference theory, meaning that as long as a feature’s reliability is reduced (or improved), observers will improve (or reduce) their reliance on the other feature to make a feature estimation.

In the current study, we conducted two experiments to examine whether serial dependencies between form orientation and motion direction were asymmetric by varying both form and motion displays’ reliabilities. If the changing trends were different between the serial effect of form orientation on the motion direction perception and the serial effect of motion direction on the form orientation when the displays’ reliabilities were varied, the serial dependences between form orientation and motion direction could be asymmetric. Additionally, varying displays’ reliability can help us answer whether the serial dependences between form and motion are consistent with the Bayesian inference account. This study not only enriches the existing studies but also improves our understanding of the mechanisms underlying the integration of form and motion features and the mechanisms underlying serial dependence.

## Experiment 1. Attractive serial dependence of motion direction also on the form orientation perception

Wang et al. (2022) found that the perceived motion direction was biased toward the previously seen form orientation, showing an attractive serial dependence of form orientation on the motion direction perception. In the first experiment of the current study, we examined the opposite trend: whether there was a serial dependence of motion direction on the form orientation perception. If yes, then the serial dependence was attractive or repulsive.

### Methods

#### Participants

Eighteen participants (9 females, 9 males, age 18–25 years) were recruited from Zhejiang Normal University. All were naïve to the experimental purpose and had normal or corrected-to-normal vision. The sample size was decided based on the previous serial dependence studies (e.g., Fischer and Whitney, 2014; Xu et al., 2022). The experiment was approved by the Scientific and Ethical Review Committee in the Department of Psychology of Zhejiang Normal University (ZSRT2022012). One participant did not complete the experiment, so the data of 17 participants were analyzed.

#### Stimuli and apparatus

Two types of stimulus displays were generated. (1) Form displays (Figure 1A, 112° H × 80° V) consisted of 45 dot pairs (diameter: 0.28°, luminance: 22.5 cd/cm^2^). The distance between two dots in each dot pair was 1°. When we connected the two dots in each dot pair, the extensions of the lines met at a point called the form focus of expansion (form FoE), indicating the form orientation (blue “×” in Figure 1A). The form orientation was randomly selected from the range of [−45°, 45°] with a step of 1°. (2) Motion displays (Figure 1B, 112° H × 80° V) simulated observers translating in a 3D dot-cloud at 1.5 m/s. The dot-cloud consisted of 90 dots (diameter: 0.28°, luminance: 22.5 cd/cm^2^; dot density: 0.01 dots/deg^2^), and its depth range was from 0.2 m to 5 m. From the observer’s view, all dots originated from one point on the screen called the motion FoE (yellow “+” in Figure 1A), indicating the motion direction. The motion direction was also randomly selected from the range of [−45°, 45°] with a step of 1°. Note that our form and motion displays were separately presented differing from the Glass pattern used in Or et al. (2010) and Pavan et al. (2017a).

The displays were programmed in MATLAB using the Psychophysics Toolbox 3 and presented on a 27-inch Dell monitor (resolution: 2560 H × 1,440 V pixels; refresh rate: 120 Hz) with NVIDIA GeForce GTX 1660Ti graphics card.

#### Procedure

The laboratory was light-excluded. Participants’ heads were stabilized with a chin-rest. The viewing distance was 20 cm. Participants viewed displays with their right eye (monocularly) to reduce the conflict between two depth cues: motion parallax (indicating a 3D moving stimulus) and binocular disparity (indicating a flat 2D display screen). Participants were asked to fixate on the display center throughout the experiment.

**Figure 2:**
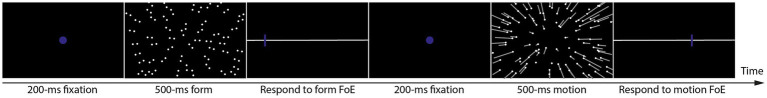
Illustration of one trial procedure.

Each trial (Figure 2) started with a 200-ms blue fixation display, followed by one 500-ms form or motion display. After each display, participants were asked to move a mouse-controlled probe to indicate their perceived position of form FoE or motion FoE along the horizontal line. After the participants’ responses, the next trial started. Each participant was asked to finish 1,320 trials (660 form and 660 motion trials). The form and motion trials were alternately presented, and the experiment always started with a form trial. In each trial, form and motion FoEs were randomly selected from the range of [−45°, 45°] with a step of 1°.

Before starting the experiment, participants were given 20 practice trials randomly selected from the experiment part. The experiment started after the practice and lasted for about 30 min.

#### Data analysis

Consistent with the previous serial dependence studies (e.g., Fischer and Whitney, 2014; Fritsche et al., 2017; Sun et al., 2020), we analyzed the data on the group level.

Several studies have found that center bias is in the motion perception (Sun et al., 2020, 2022; Xu et al., 2022). However, it does not know that the size of center bias is constant or linearly or nonlinearly changed with increasing the motion direction. Therefore, it is unreasonable to fit the perceived motion direction as a linear function of the actual motion direction and attribute the predicted perceived motion direction to center bias (Sun et al., 2020). As a result, it can be questioned that the difference between the predicted and actual perceived motion direction (i.e., the residual motion error) is from the serial dependence. To avoid this question, we fitted the perceived motion direction ($\theta_{FL}$) as a multi-factor linear function of the motion direction of the current (*n*th, *n* = 2, 3, etc.) trial ($\theta_{\text{curr\_FL}}$), the form orientations of the previous 1st (*n*-1st) trial ($\theta_{\text{pre\_FM}}$) and the next (*n* + 1st) trial ($\theta_{\text{next\_FM}}$), given by (also see Zhang and Luo, 2023):


(1) $$\begin{array}{l} \hat{\theta_{FL}}=b_{\text{curr\_FL}}\theta_{\text{curr\_FL}}+b_{\text{pre\_FM}}\theta_{\text{pre\_FM}}\\ \;\;\;\;\;\;\;\;\;+b_{\text{next\_FM}}\theta_{\text{next\_FM}}+\varepsilon \end{array}$$

in which, $\hat{\theta_{FL}}$ is the predicted perceived motion direction, $b_{\text{curr\_FL}}$, $b_{\text{pre\_FM}}$, and $b_{\text{next\_FM}}$ are the slopes of different factors. ε is the constant. If $b_{\text{curr\_FL}}$ is equal to 1, the perceived motion direction is the same as the actual motion direction, showing a perfect performance. The more $b_{\text{curr\_FL}}$ deviated from 1, the lower the accuracy of the perceptual performance. Especially, if $b_{\text{curr\_FL}}$ is smaller than 1, the perceived motion direction was systematically compressed toward the center (0°), indicating a center bias is in the motion direction perception; if there is a serial dependence of form orientation on the motion direction perception, the effect of $b_{\text{pre\_FM}}$ will be significant but the effect of $b_{\text{next\_FM}}$ will be insignificant. In addition, a positive $b_{\text{pre\_FM}}$ indicates an attractive serial dependence, meaning that the current motion direction perception is biased toward the previous form orientation; whereas a negative $b_{\text{pre\_FM}}$ indicates a repulsive serial dependence, meaning that the current motion direction perception is repelled from the previous form orientation.

Next, we fitted the perceived form orientation ($\theta_{FM}$) as a multi-factor linear function of the form orientation of the current (*n*th, *n* = 2, 3, etc.) trial ($\theta_{\text{curr\_FM}}$), the motion directions of the previous 1st (*n*-1st) trial ($\theta_{\text{pre\_FL}}$) and the next (*n* + 1^st^) trial ($\theta_{\text{next\_FL}}$), given by:


(2) $$\begin{array}{l} \hat{\theta_{FM}}=b_{\text{curr\_FM}}\theta_{\text{curr\_FM}}+b_{\text{pre\_FL}}\theta_{\text{pre\_FL}}\\ \;\;\;\;\;\;\;\;\;\;\;+b_{\text{next\_FL}}\theta_{\text{next\_FL}}+\varepsilon \end{array}$$

in which, $\hat{\theta_{FM}}$ is the predicted perceived form orientation, $b_{\text{curr\_FM}}$, $b_{\text{pre\_FL}}$, and $b_{\text{next\_FL}}$ are the slopes of different factors. ε is the constant. Similarly, if center bias is in the form orientation, $b_{\text{curr\_FM}}$ will be smaller than 1; if there is a serial dependence of motion direction on the form orientation perception, the effect of $b_{\text{pre\_FL}}$ will be significant but the effect of $b_{\text{next\_FL}}$ will be insignificant. In addition, a positive $b_{\text{pre\_FL}}$ indicates an attractive serial dependence, meaning that the current form orientation perception is biased toward the previous motion direction; whereas a negative $b_{\text{pre\_FL}}$ indicates a repulsive serial dependence, meaning that the current form orientation perception is repelled from the previous motion direction.

### Results and discussion

**Figure 3:**
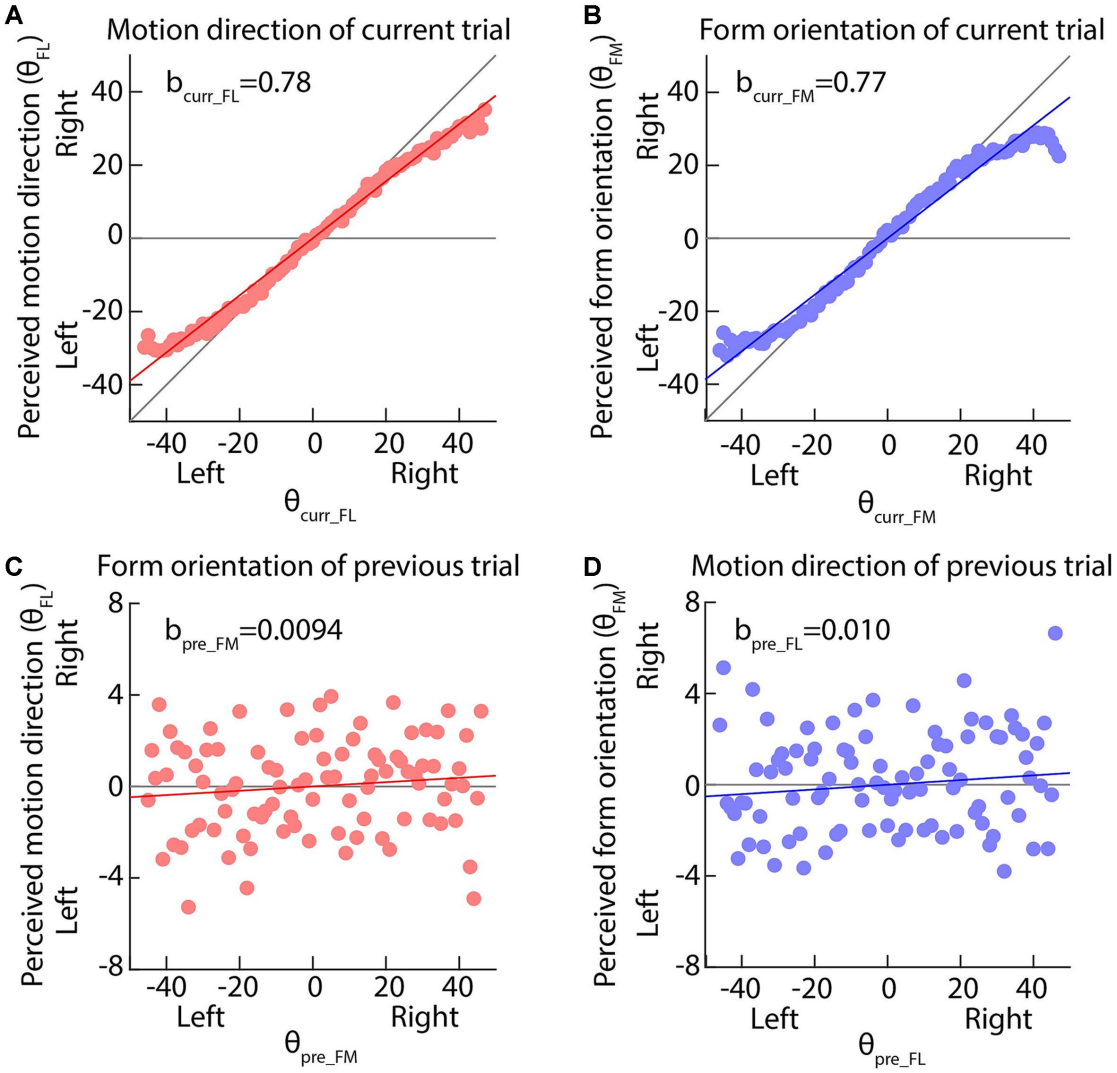
**(A,B)** The perceived motion direction or form orientation is against the actual feature values of current trials. The solid red or blue lines indicate the best fitting results of our multi-factor linear functions (Equations 1, 2). The diagonal and horizontal gray lines indicate the perfect performance and pure center bias. **(C,D)** The perceived motion direction or form orientation is against the actual feature values of previous trials. The horizontal gray line indicates no effect of previous feature on the current perception. In all panels, dots are the average perceived values across all participants; the solid red or blue lines indicate the best fitting results of our multi-factor linear functions (Equations 1, 2).

Figures 3A,B plots the perceived motion direction (form orientation) against the actual motion direction (actual form orientation) of the current trial, clearly showing that the perceived values are compressed toward the display center (horizontal gray line). Table 1 clearly indicates that $b_{\text{curr\_FL}}$ and $b_{\text{curr\_FM}}$ are significantly larger than 0 (*p*s < 0.001) and the upper bands of the 95% CIs of $b_{\text{curr\_FL}}$ and $b_{\text{curr\_FM}}$ are smaller than 1, indicating that the perceptions of motion direction and form orientation are center biased.

**Table 1:** Results of linear function between the current (*n*th, *n* = 2, 3, etc.) trial, the previous 1st (*n*-1st) trial and the next (*n* + 1st) trial (Equations 1, 2).

	Equation (1)			Equation (2)		
	$b_{\text{curr\_FL}}$	$b_{\text{pre\_FM}}$	$b_{\text{next\_FM}}$	$b_{\text{curr\_FM}}$	$b_{\text{pre\_FL}}$	$b_{\text{next\_FL}}$
b	0.78***	0.0094*	−0.0012	0.77***	0.010*	0.0011
SE	0.0037	0.0037	0.0037	0.0046	0.0046	0.0046
95% CI	0.77 0.79	0.0022 0.017	−0.0083 0.0059	0.76 0.78	0.0011 0.020	−0.0079 0.010

The significant level of each coefficient is the result of *t*-test in linear regression analysis that indicates whether the coefficient was significantly different from 0. *p < 0.05; **p < 0.01; ***p < 0.001.

Importantly, Figures 3C,D plots the perceived motion direction (form orientation) against the form orientation (motion direction) of the previous trial, clearly showing that the perceived values are biased toward the previous seen features. Both $b_{\text{pre\_FM}}$ and $b_{\text{pre\_FL}}$ are significantly larger than 0 (*p*s < 0.01) and neither $b_{\text{next\_FM}}$ nor $b_{\text{next\_FL}}$ is significant (*p*s > 0.079), suggesting that the current motion direction perception was biased toward the previously seen form orientation and the current form orientation perception was biased toward the previously seen motion direction. That is, a bidirectional attractive serial dependence was between motion direction and form orientation perceptions. The current experiment further complemented the findings of Wang et al. (2022).

In the current experiment, although both motion and form displays contained 90 dots, it could not guarantee that the two displays’ reliabilities were identical. Hence, a direct comparison of the slopes of the two serial dependencies was unconvincing for examining the symmetry of the serial dependencies between form orientation and motion direction. To address this question, we varied the dot densities of motion and form displays in Experiment 2, which theoretically directly changed the stimuli’s internal and external reliabilities. We compared the changing trends of the serial dependencies between form orientation and motion direction when the reliabilities of motion and form display changed. If the changing trends differed, serial dependencies between form orientation and motion direction were asymmetric.

## Experiment 2. Varying the displays’ dot density reveals the asymmetric serial dependencies between form and motion

Several studies proposed that serial dependence was consistent with the Bayesian inference theory (Cicchini et al., 2018; van Bergen and Jehee, 2019; Xu et al., 2022). These studies showed that when the reliability of the current feature was reduced, participants would rely more on previous features. However, they ignored the effects of changing the reliability of previous features on the current feature perception. Three studies pointed out that the size of serial dependence only depended on the reliability of current features (Cicchini et al., 2018; Ceylan et al., 2021; Gallagher and Benton, 2022). According to the bidirectional proposal of the Bayesian inference theory, serial dependence was a partial Bayesian inference process.

The current experiment examined whether the serial dependencies between form orientation and motion direction were consistent with the Bayesian inference theory by varying the two displays’ dot densities. In addition, we examined whether the serial dependencies between form orientation and motion direction were symmetric by comparing the changing trends of the serial dependencies. If changing trends significantly differed, the serial dependences between form orientation and motion direction were asymmetric.

### Methods

#### Participants

Two groups of new participants from Zhejiang Normal University were enrolled. Each group had 18 participants (Group 1: 10 females, eight males; age, 18–25 years; Group 2: 10 females, eight males; age, 18–25 years). All were naïve to the experimental purpose and had normal or corrected-to-normal vision. The sample size was decided based on the previous serial dependence studies (e.g., Fischer and Whitney, 2014; Xu et al., 2022). The experiment was approved by the Scientific and Ethical Review Committee in the Department of Psychology of Zhejiang Normal University (ZSRT2022012).

#### Stimuli, apparatus, and procedures

The stimuli, apparatus and procedures were similar to those in Experiment 1, except that (1) participants of Group 1 were shown form displays with 90 dots (Figure 1A, high dot density, dot density: 0.01 dots/deg^2^), but the motion displays were either with 28 dots (Figure 1D, low dot density, dot density: 0.003 dots/deg^2^) or 90 dots (Figure 1B, high dot density, dot density: 0.01 dots/deg^2^). (2) Participants of Group 2, were shown motion displays with 90 dots (Figure 1B, high dot density, dot density: 0.01 dots/deg^2^), but the form displays were either with 14 dot pairs (Figure 1C, low dot density, dot density: 0.003 dots/deg^2^) or 45 dot pairs (Figure 1A, high dot density, dot density: 0.01 dots/deg^2^). The displays’ reliabilities decreased with the decrease in the displays’ dot densities. (3) Each participant was asked to finish two blocks of trials. Each block corresponded to one experimental condition: Group 1, form with high density and motion with low density, form with high density and motion with high density; Group 2, motion with high density and form with low density, and motion with high density and form with high density. (4) Each participant took about 70 min to finish the experiment.

#### Data analysis

The data analysis methods were similar to the Experiment 1. Note that if $b_{\text{curr\_FL}}$ and $b_{\text{curr\_FM}}$ are equal to 1, the perceived feature values are the same as the actual feature values, showing a perfect performance. The more $b_{\text{curr\_FL}}$ and $b_{\text{curr\_FM}}$ deviated from 1, the lower the accuracy of the perceptual performance, showing that the stimuli became unreliable.

In addition, permutation tests were conducted to examine whether the reliability of displays increased with increasing the dot-density and whether the changing trends of serial dependencies between form orientation and motion direction were the same when the two displays’ reliabilities were varied. Specifically, we grouped the data of low and high dot-density conditions. The data were shuffled 10,000 times. In each shuffle iteration, for Equation (1), we randomized the perceived motion direction and the form orientations of previous and next trials; for Equation (2), we randomized the perceived form orientation and the motion directions of previous and next trials. Then, half of the shuffled data were assigned to the low-density condition; the left data were assigned to the high-density condition. Equations (1, 2) were fitted with the shuffled data, resulting in to shuffled coefficients. Next, we calculated the difference in the shuffled coefficient between the low and high dot densities, generating a null distribution for each coefficient difference. Finally, we calculated the proportion of the coefficient difference larger than the absolute value of the coefficient difference of participants’ data (two-tailed test). The proportion was taken as the *p* value. If the p value is smaller than 0.05, the difference in the coefficient between low and high dot-density conditions is significant.

### Results and discussion

**Figure 4:**
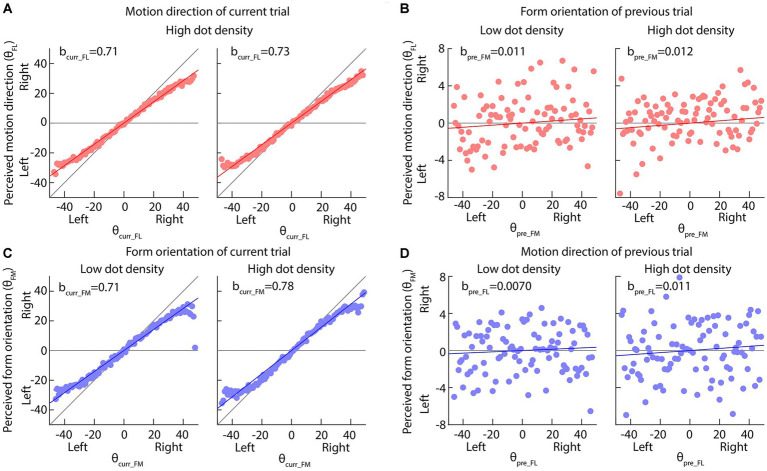
Results of Experiment 2 when the dot-density of form displays was varied but that of motion displays was fixed. **(A,C)** The perceived motion direction or form orientation is against the actual feature values of current trials. The solid red or blue lines indicate the best fitting results of our multi-factor linear functions (Equations 1, 2). The diagonal and horizontal gray lines indicate the perfect performance and pure center bias. **(B,D)** The perceived motion direction or form orientation is against the actual feature values of previous trials. The horizontal gray line indicates no effect of previous feature on the current perception. In all panels, dots are the average perceived values across all participants; the solid red or blue lines indicate the best fitting results of our multi-factor linear functions (Equations 1, 2).

**Figure 5:**
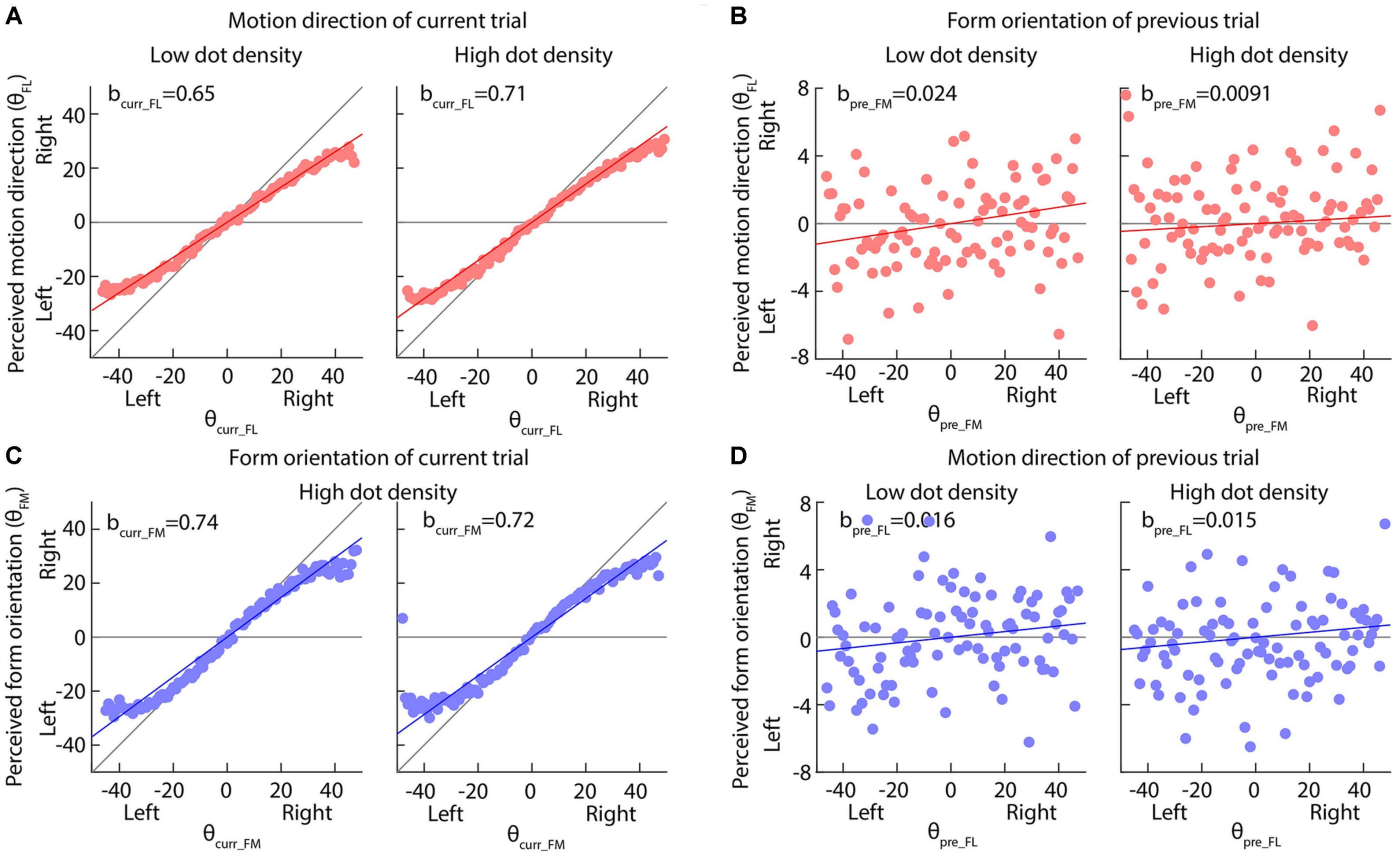
Results of Experiment 2 when the dot-density of motion displays was varied but that of the form displays were fixed. **(A,C)** The perceived motion direction or form orientation is against the actual feature values of current trials. The solid red or blue lines indicate the best fitting results of our multi-factor linear functions (Equations 1, 2). The diagonal and horizontal gray lines indicate the perfect performance and pure center bias. **(B,D)** The perceived motion direction or form orientation is against the actual feature values of previous trials. The horizontal gray line indicates no effect of previous feature on the current perception. In all panels, dots are the average perceived values across all participants; the solid red or blue lines indicate the best fitting results of our multi-factor linear functions (Equations 1, 2).

Figures 4A,C, 5A,C and Tables 2, 3 show the results of our two linear functions when the dot-densities of the form and motion displays are varied. Consistent with Experiment 1, $b_{\text{curr\_FL}}$ and $b_{\text{curr\_FM}}$ were all significantly different from 0 (*p*s < 0.001) and smaller than 1 (upper bands of 95% CIs are smaller than 1), so there is center bias in the perceptions of motion direction and form orientation. $b_{\text{pre\_FM}}$ and $b_{\text{pre\_FL}}$ are generally significantly larger than 0 (*p*s < 0.01, except the $b_{\text{pre\_FL}}$ in the low-density form display condition, *p* = 0.19), suggesting attractive serial dependencies were between form orientation and motion direction.

**Table 2:** Results of linear function between the current (*n*th, *n* = 2, 3, etc.) trial, the previous 1st (*n*-1st) trial and the next (*n* + 1st) trial (Equation 1) when the dot-density of the form display was varied.

Form display		Equation (1)			Equation (2)		
		$b_{\text{curr\_FL}}$	$b_{\text{pre\_FM}}$	$b_{\text{next\_FM}}$	$b_{\text{curr\_FM}}$	$b_{\text{pre\_FL}}$	$b_{\text{next\_FL}}$
Low-density	b	0.71***	0.011**	−0.0037	0.71***	0.0070	0.0057
	SE	0.0037	0.0036	0.0036	0.0054	0.0053	0.0053
	95% CI	0.71 0.72	0.0041 0.0184	−0.011 0.0034	0.70 0.72	−0.0037 0.0171	−0.0047 0.0164
High-density	b	0.73***	0.012***	−0.0066	0.78***	0.011*	−0.0030
	SE	0.0035	0.0034	0.0034	0.0050	0.0050	0.0050
	95% CI	0.72 0.74	0.0057 0.019	−0.013 0.0001	0.77 0.79	0.0013 0.021	−0.013 0.0066

The significant level of each coefficient is the result of *t*-test in linear regression analysis that indicates whether the coefficient is significantly different from 0. *p < 0.05; **p < 0.01; ***p < 0.001.

**Table 3:** Results of linear function between the current (*n*th, *n* = 2, 3, etc.) trial, the previous 1st (*n*-1st) trial and the next (*n* + 1st) trial (Equations 1, 2) when the dot-density of the motion display was varied.

Motion display		Equation (1)			Equation (2)		
		$b_{\text{curr\_FL}}$	$b_{\text{pre\_FM}}$	$b_{\text{next\_FM}}$	$b_{\text{curr\_FM}}$	$b_{\text{pre\_FL}}$	$b_{\text{next\_FL}}$
Low-density	b	0.65***	0.024***	−0.0015	0.74***	0.016**	0.0060
	SE	0.0043	0.0043	0.0043	0.0051	0.0051	0.0051
	95% CI	0.64 0.66	0.016 0.033	−0.0097 0.0066	0.72 0.75	0.0066 0.027	−0.0040 0.016
High-density	b	0.71***	0.0091*	0.0034	0.72***	0.015**	0.0014
	SE	0.0041	0.0040	0.0040	0.0052	0.0052	0.0052
	95% CI	0.70 0.72	0.0013 0.017	−0.0046 0.011	0.70 0.73	0.0043 0.025	−0.0089 0.012

The significant level of each coefficient is the result of t-test in linear regression analysis that indicates whether the coefficient was significantly different from 0. *p < 0.05; **p < 0.01; ***p < 0.001.

When the dot density of the form display was increased, the $b_{\text{curr\_FM}}$ was increased (Mean ± SE: 0.71 ± 0.0054 in low dot density 0.78 ± 0.0050 in high dot density; Permutation test: *p* value = 0.043) (Left and right panels in Figure 4C), suggesting that the size of center bias in the form orientation perception decreased and the perception became more accurate. Similar results were also observed when the dot density of the motion display was increased ($b_{\text{curr\_FL}}$, Mean ± SE: 0.65 ± 0.0043 in low dot density 0.71 ± 0.0041 in high dot density; Permutation test: *p* value = 0.043; *p* value <0.001) (Left and right panels in Figure 5A). These results suggested that increasing dot density of the form and motion displays indeed increased the two displays’ reliability.

Neither the reliability of the current form display nor that of the previous motion display affected the serial dependence of motion direction on the form orientation perception ($b_{\text{pre\_FL}}$, Permutation test: *p* values >0.34) (Figures 4D, 5D), inconsistent with Bayesian inference account in the current experimental set-up.

However, the serial dependence of form orientation on the motion direction perception ($b_{\text{pre\_FM}}$, Permutation test: *p* value = 0.016) was affected by the reliability of the current motion display (Figure 5B) rather than that of the previous form display (Figure 4B) (*p* value = 0.54), consistent with the partial Bayesian inference account (Ceylan et al., 2021; Gallagher and Benton, 2022).

Taken together, serial dependencies between form orientation and motion direction showed different trends when the reliabilities of form and motion displays were varied. Hence, the serial dependencies between form orientation and motion direction were asymmetric.

## General discussion

In the current study, we conducted two experiments to examine whether the form orientation and motion direction could be integrated across the temporal domain, showing serial dependencies. On this basis, we further investigated the symmetry of the serial dependencies between form orientation and motion direction and whether they were consistent with the Bayesian inference theory. Our results showed attractive serial dependencies between form orientation and motion direction. When the reliabilities of motion or form displays were changed, the changing trends of the serial dependencies between form orientation and motion direction were inconsistent (Experiment 2), suggesting that the serial dependencies between form orientation and motion direction were asymmetric. Moreover, according to the Bayesian inference theory, when one feature’s reliability is reduced, observers would rely more on other features to make an estimation, which is a bidirectional process. However, the serial dependence of form orientation on the motion direction perception was only affected by the reliability of the current motion display, consistent with a partial Bayesian inference account (Ceylan et al., 2021; Gallagher and Benton, 2022). Neither the reliability of the previous motion display nor that of the current form display affected the serial dependence of motion direction on the form orientation perception, inconsistent with the Bayesian inference account.

### From concurrent integration to cross-temporal integration (serial dependence)

If taking Geisler (1999) as the first study that systematically examined the integration of form and motion, we deducted that more than 95% of related studies focused on the integration mechanisms of two information concurrently presented in the past 24 years (see Donato et al., 2020 for reviews; Krekelberg et al., 2003; Edwards and Crane, 2007; Niehorster et al., 2010; Mather et al., 2013; Day and Palomares, 2014; Kuai et al., 2020; Donato et al., 2022). Tang et al. (2015) found that the perceived motion direction was biased toward the previously seen form orientation. However, each form orientation was presented for 3 s in their study, which induced form orientation aftereffects. Due to the form aftereffect when the motion display was presented, their findings reflected the mechanisms underlying the concurrent integration of motion direction and form orientation. To eliminate the form aftereffect, Wang et al. (2022) reduced the presentation time of the previous form display (i.e., 500 ms). They found that the perceived motion direction was biased toward the previous form orientation, revealing the serial dependence of form orientation on the motion direction perception. However, Wang’s study did not examine the serial dependence of motion direction on the form orientation perception. Therefore, inspired by their work, our current study not only replicated their attractive serial dependence of form orientation on the motion direction perception but also found an attractive serial dependence of motion direction on the form orientation perception. That is, we revealed the mutual serial dependencies between motion direction and form orientation.

### Enriching the studies about serial dependencies between different features

Additionally, our current study extended the studies on serial dependencies between different features. Before our current study, Fornaciai and Park (2019a) first found serial effects between visual flash counting and numerosity. After this study, others further revealed serial dependencies between dot-array orientations and Gabor orientations (strictly speaking, they could be the same features with different visual representations) (Ceylan et al., 2021), between time and numerosity (Togoli et al., 2021), and between emotion sounds and emotion images (Van der Burg et al., 2021). Moreover, after Wang et al. (2022), we found the bidirectional serial dependencies between form orientation and motion direction. The serial dependencies between different features suggested that the serial dependence occurred at either the perceptual-integration stage or the post-perceptual decision/memory stages (see also Fornaciai and Park, 2019b, 2021; Ceylan et al., 2021).

In addition, by varying the displays’ reliability, we found that the serial dependencies between form orientation and motion direction showed different changing trends, suggesting that the serial dependencies between form orientation and motion direction were asymmetric. To our knowledge, the current study was the first one revealing the asymmetric across-temporal integration between different features, inspiring us to examine other features further.

### Challenging the effectiveness of the Bayesian inference account in serial dependence

Cicchini et al. (2018) first proposed that the Bayesian inference theory could explain serial dependence. However, in this study, the reliabilities of current and previous features were positively correlated. Although a Bayesian model well explained their results, they did not rigorously control the reliability difference between the current and previous features. Hence, Ceylan et al. (2021) sequentially showed participants a series of oriented Gabor patches with different spatial frequencies. The higher the spatial frequency, the more reliable the Gabor patch. They found that when the reliability of current stimuli was reduced while the previous was stable, observers showed a stronger serial dependence of previous stimuli on the current perception. However, when the reliability of previous stimuli was reduced while the current was stable, the serial dependence was not significantly changed. Therefore, the serial dependence was partially consistent with the Bayesian inference account (Gallagher and Benton, 2022). The serial dependence of form orientation on the motion direction perception well matched their findings. However, the serial dependence of motion direction on the form orientation perception was not affected by the reliabilities of previous motion and current form displays, inconsistent with Bayesian inference theory. These findings inspired us to think carefully about the computational mechanisms of serial dependence.

### Additional points

We found that serial dependencies between motion direction and form orientation were asymmetric, indicating that the neurons responded to the two features differently. Notably, the motion direction and form orientation used in the current study are global features, meaning that participants have to sum up several local features to get the motion direction and form orientation. Therefore, the receptive field of these neurons should be large enough to integrate several local inputs. According to the physiological studies, these neurons can be located at the cortical areas MT/V5, MST, V3B/KO (see review: Kourtzi et al., 2008; Donato et al., 2020; experimental studies, e.g., Kourtzi and Kanwisher, 2000; Kourtzi et al., 2002; Kuai et al., 2020). However, there are some cortical areas, such as V1/V2 (Krekelberg et al., 2005; Sincich and Horton, 2005; Lu et al., 2010; Wattam-Bell et al., 2010), V4 (Ferrera and Maunsell, 2005; Tolias et al., 2005; Handa et al., 2010, 2017; Handa and Mikami, 2018) that have small receptive fields and mainly process the local form and motion features. Therefore, the asymmetric serial dependencies between global form and motion displays in our current study do not imply asymmetric serial dependencies between local form and motion displays. In future studies, we should adopt the local form and motion displays in which all dots consistently move along the same direction and the orientations of all dot pairs are parallel (e.g., Or et al., 2010; Pavan et al., 2017a), to re-examine our asymmetric serial dependencies between form and motion features.

Moreover, some readers could argue that the asymmetric serial dependencies between form orientation and motion direction could be due to the different impact of the dot density manipulation on the reliability of the form and motion features. That is true. However, our data showed that the reliabilities of the form and motion were evidently decreased with decreasing dot density. We found that the serial dependence of form orientation on the perception of motion direction was partially consistent with Bayesian inference theory. In contrast, the serial dependence of motion direction on the perception of form orientation was not consistent with the Bayesian inference theory. This directly shows the asymmetric serial dependencies between the two features. Future studies can consider use more directly methods (e.g., neurophysiological techniques) to re-examine our findings and figure out the mechanisms underlying the asymmetry.

### Summary

In conclusion, our current study found that serial dependencies between form orientation and motion direction could be asymmetric and were partially consistent with the Bayesian inference theory. These findings suggested that the cross-temporal and concurrent integrations of form and motion differed, prompting us to explore the mechanisms underlying the serial dependence between form orientation and motion direction from the computational and neurophysiological perspectives.

## Data availability statement

The raw data supporting the conclusions of this article will be made available by the authors, without undue reservation.

## Ethics statement

The studies involving humans were approved by the Scientific and Ethical Review Committee in the Department of Psychology of Zhejiang Normal University. The studies were conducted in accordance with the local legislation and institutional requirements. The participants provided their written informed consent to participate in this study.

## Author contributions

QS designed the study, analyzed the data, and wrote the paper. F-HY and X-MG collected the data and proofread the paper. All authors contributed to the article and approved the submitted version.

## Funding

This study was supported by National Natural Science Foundation of China, China (No. 32200842) to QS.

## Conflict of interest

The authors declare that the research was conducted in the absence of any commercial or financial relationships that could be construed as a potential conflict of interest.

## Publisher’s note

All claims expressed in this article are solely those of the authors and do not necessarily represent those of their affiliated organizations, or those of the publisher, the editors and the reviewers. Any product that may be evaluated in this article, or claim that may be made by its manufacturer, is not guaranteed or endorsed by the publisher.

## References

[ref3] Bernardo J. M. Smith A. F. M. (1994). Bayesian theory. New York: Wiley.

[ref4] Caelli T. Brettel H. Rentschler I. Hilz R. (1983). Discrimination thresholds in the two-dimensional spatial frequency domain. Vis. Res. 23, 129–133. doi: 10.1016/0042-6989(83)90135-9, PMID: 6868387

[ref5] Ceylan G. Herzog M. H. Pascucci D. (2021). Serial dependence does not originate from low-level visual processing. Cognition 212, 104707–104709. doi: 10.1016/j.cognition.2021.104709 33838523

[ref6] Cicchini G. M. Mikellidou K. Burr D. C. (2018). The functional role of serial dependence. Proc. R. Soc. B 285:20181722. doi: 10.1098/rspb.2018.1722 PMC6235035 30381379

[ref7] Cox R. T. (1946). Probability, frequency and reasonable expectation. Am. J. Phys. 14, 1–13. doi: 10.1119/1.1990764

[ref8] Day A. M. Palomares M. (2014). How temporal frequency affects global form coherence in Glass patterns. Vis. Res. 95, 18–22. doi: 10.1016/j.visres.2013.11.009, PMID: 24325849

[ref9] De Gardelle V. Kouider S. Sackur J. (2010). An oblique illusion modulated by visibility: non-monotonic sensory integration in orientation processing. J. Vis. 10, 1–9. doi: 10.1167/10.10.6, PMID: 20884471

[ref10] DeYoe E. A. Van Essen D. C. (1988). Concurrent processing streams in monkey visual cortex. Trends Neurosci. 11, 219–226. doi: 10.1016/0166-2236(88)90130-0 2471327

[ref11] Donato R. Pavan A. Campana G. (2020). Investigating the interaction between form and motion processing: a review of basic research and clinical evidence. Front. Psychol. 11:566848. doi: 10.3389/fpsyg.2020.566848, PMID: 33192845 PMC7661965

[ref12] Donato R. Pavan A. Cavallin G. Ballan L. Betteto L. Nucci M.. (2022). Mechanisms underlying directional motion processing and form-motion integration assessed with visual perceptual learning. Vision 6:29. doi: 10.3390/vision6020029, PMID: 35737415 PMC9229663

[ref13] Edwards M. Crane M. F. (2007). Motion streaks improve motion detection. Vis. Res. 47, 828–833. doi: 10.1016/j.visres.2006.12.005, PMID: 17258262

[ref14] Ferrera V. P. Maunsell J. H. (2005). Motion processing in macaque V4. Nat. Neurosci. 8:1125. doi: 10.1038/nn0905-1125a 16127440

[ref15] Fischer J. Whitney D. (2014). Serial dependence in visual perception. Nat. Neurosci. 17, 738–743. doi: 10.1038/nn.3689, PMID: 24686785 PMC4012025

[ref17] Fornaciai M. Park J. (2019a). Serial dependence generalizes across different stimulus formats, but not different sensory modalities. Vis. Res. 160, 108–115. doi: 10.1016/j.visres.2019.04.011, PMID: 31078663

[ref18] Fornaciai M. Park J. (2019b). Spontaneous repulsive adaptation in the absence of attractive serial dependence. J. Vis. 19:21. doi: 10.1167/19.5.21 31112999

[ref19] Fornaciai M. Park J. (2021). Disentangling feedforward versus feedback processing in numerosity representation. Cortex 135, 255–267. doi: 10.1016/j.cortex.2020.11.013, PMID: 33412370

[ref20] Fritsche M. Mostert P. de Lange F. P. (2017). Opposite effects of recent history on perception and decision. Curr. Biol. 27, 590–595. doi: 10.1016/j.cub.2017.01.006, PMID: 28162897

[ref22] Gallagher G. K. Benton C. P. (2022). Stimulus uncertainty predicts serial dependence in orientation judgements. J. Vis. 22, 6–14. doi: 10.1167/jov.22.1.6, PMID: 35019954 PMC8762691

[ref23] Geisler W. S. (1999). Motion streaks provide a spatial code for motion direction. Nature 400, 65–69. doi: 10.1038/21886, PMID: 10403249

[ref24] Glass L. (1969). Moiré effect from random dots. Nature 223, 578–580. doi: 10.1038/223578a0, PMID: 5799528

[ref25] Goodale M. A. Milner A. (1992). Separate visual pathways for perception and action. Trends Neurosci. 15, 20–25. doi: 10.1016/0166-2236(92)90344-8 1374953

[ref26] Handa T. Inoue M. Mikami A. (2010). Neuronal activity during discrimination of shapes defined by motion in area V4. Neuroreport 21, 532–536. doi: 10.1097/WNR.0b013e3283393a5f, PMID: 20386346

[ref27] Handa T. Mikami A. (2018). Neuronal correlates of motion-defined shape perception in primate dorsal and ventral streams. Eur. J. Neurosci. 48, 3171–3185. doi: 10.1111/ejn.14121, PMID: 30118167

[ref28] Handa T. Unno S. Mikami A. (2017). Temporal property of single-cell activity in response to motion-defined shapes in monkey dorsal and ventral cortical areas. Neuroreport 28, 793–799. doi: 10.1097/WNR.0000000000000826, PMID: 28678113

[ref29] Jaynes E. T. (1986). “Bayesian methods: general background” in Maximum entropy and Bayesian methods in applied statistics. ed. Justice J. H. (Cambridge, UK: Cambridge University Press)

[ref30] Kiyonaga A. Scimeca J. M. Bliss D. P. Whitney D. (2017). Serial dependence across perception, attention, and memory. Trends Cogn. Sci. 21, 493–497. doi: 10.1016/j.tics.2017.04.011, PMID: 28549826 PMC5516910

[ref31] Kourtzi Z. Bülthoff H. H. Erb M. Grodd W. (2002). Object-selective responses in the human motion area MT/MST. Nat. Neurosci. 5, 17–18. doi: 10.1038/nn780, PMID: 11740503

[ref32] Kourtzi Z. Kanwisher N. (2000). Activation in human MT/MST by static images with implied motion. J. Cogn. Neurosci. 12, 48–55. doi: 10.1162/08989290051137594, PMID: 10769305

[ref33] Kourtzi Z. Krekelberg B. van Wezel R. J. A. (2008). Linking form and motion in the primate brain. Trends Cogn. Sci. 12, 230–236. doi: 10.1016/j.tics.2008.02.013 18468943

[ref34] Krekelberg B. Dannenberg S. Hoffmann K. P. Bremmer F. Ross J. (2003). Neural correlates of implied motion. Nature 424, 674–677. doi: 10.1038/nature01852 12904793

[ref35] Krekelberg B. Vatakis A. Kourtzi Z. (2005). Implied motion from form in the human visual cortex. J. Neurophysiol. 94, 4373–4386. doi: 10.1152/jn.00690.2005, PMID: 16107528

[ref36] Kuai S. G. Chen J. Xu Z. X. Li J. M. Field D. T. Li L. (2020). Integration of motion and form cues for the perception of self-motion in the human brain. J. Neurosci. 40, 1120–1132. doi: 10.1523/JNEUROSCI.3225-18.2019, PMID: 31826945 PMC6988997

[ref37] Liu L. Wang F. Zhou K. Ding N. Luo H. (2017). Perceptual integration rapidly activates dorsal visual pathway to guide local processing in early visual areas. PLoS Biol. 15:e2003646. doi: 10.1371/journal.pbio.2003646, PMID: 29190640 PMC5726727

[ref38] Lu H. D. Chen G. Tanigawa H. Roe A. W. (2010). A motion direction map in macaque V2. Neuron 68, 1002–1013. doi: 10.1016/j.neuron.2010.11.020, PMID: 21145011 PMC3391546

[ref39] MacKay D. J. C. (2003). Information theory, inference and learning algorithms. Cambridge, UK: Cambridge University Press.

[ref40] Mather G. Pavan A. Bellacosa Marotti R. Campana G. Casco C. (2013). Interactions between motion and form processing in the human visual system. Front. Comput. Neurosci. 7, 1–6. 23730286 10.3389/fncom.2013.00065 PMC3657629

[ref41] Matsuyoshi D. Hirose N. Mima T. Fukuyama H. Osaka N. (2007). Repetitive transcranial magnetic stimulation of human MT+ reduces apparent motion perception. Neurosci. Lett. 429, 131–135. doi: 10.1016/j.neulet.2007.10.002, PMID: 17997041

[ref42] Milner A. D. Goodale M. A. (2006). The visual brain in action (2nd ed.). New York: Oxford University Press.

[ref43] Mishkin M. Ungerleider L. G. Macko K. A. (1983). Object vision and spatial vision: two cortical pathways. Trends Neurosci. 6, 414–417. doi: 10.1016/0166-2236(83)90190-X

[ref44] Niehorster D. C. Cheng J. C. Li L. (2010). Optimal combination of form and motion cues in human heading perception. J. Vis. 10, 1–15. doi: 10.1167/10.11.20, PMID: 20884515

[ref45] Or C. C. Khuu S. K. Hayes A. (2010). Moving Glass patterns: asymmetric interaction between motion and form. Perception 39, 447–463. doi: 10.1068/p5917, PMID: 20514995

[ref47] Pavan A. Bimson L. M. Gall M. G. Ghin F. Mather G. (2017a). The interaction between orientation and motion signals in moving oriented Glass patterns. Vis. Neurosci. 34, 1–9. doi: 10.1017/S0952523817000086 28965515

[ref48] Pavan A. Ghin F. Donato R. Campana G. Mather G. (2017b). The neural basis of form and form-motion integration from static and dynamic translational Glass patterns: a rTMS investigation. NeuroImage 157, 555–560. doi: 10.1016/j.neuroimage.2017.06.036, PMID: 28633972

[ref49] Sincich L. C. Horton J. C. (2005). Input to V2 thin stripes arises from V1 cytochrome oxidase patches. J. Neurosci. 25, 10087–10093. doi: 10.1523/JNEUROSCI.3313-05.2005, PMID: 16267215 PMC6725776

[ref50] Sun Q. Yan R. Wang J. Li X. (2022). Heading perception from optic flow is affected by heading distribution. i-Perception 13, 1–17. doi: 10.1177/2041669522113340 PMC9706071 36457854

[ref51] Sun Q. Zhang H. Alais D. Li L. (2020). Serial dependence and center bias in heading perception from optic flow. J. Vis. 20, 1–15. doi: 10.1167/jov.20.10.1, PMID: 33001176 PMC7545086

[ref52] Tang M. F. Dickinson J. E. Visser T. A. Badcock D. R. (2015). The broad orientation dependence of the motion streak aftereffect reveals interactions between form and motion neurons. J. Vis. 15, 4–18. doi: 10.1167/15.13.4, PMID: 26381835

[ref53] Togoli I. Fedele M. Fornaciai M. Bueti D. (2021). Serial dependence in time and numerosity perception is dimension-specific. J. Vis. 21, 6–15. doi: 10.1167/jov.21.5.6, PMID: 33956059 PMC8107483

[ref54] Tolias A. S. Keliris G. A. Smirnakis S. M. Logothetis N. K. (2005). Neurons in macaque area V4 acquire directional tuning after adaptation to motion stimuli. Nat. Neurosci. 8, 591–593. doi: 10.1038/nn1446, PMID: 15834417

[ref55] Ungerleider L. G. Mishkin M. (1982). “Two cortical visual systems” in Analysis of visual behavior. eds. Goodale M. Ingle D. J. Mansfield R. J. W. (Cambridge, MA: MIT Press)

[ref56] van Bergen R. S. Jehee J. F. (2019). Probabilistic representation in human visual cortex reflects uncertainty in serial decisions. J. Neurosci. 39, 8164–8176. doi: 10.1523/JNEUROSCI.3212-18.2019, PMID: 31481435 PMC6786811

[ref9001] Van der Burg E. Toet A. Brouwer A. M. Van Erp J. B. (2021). Serial dependence of emotion within and between stimulus sensory modalities. Multisens. Res. 35, 151–172. doi: 10.1163/22134808-bja10064 34592713

[ref57] Wang X. Y. Gong X. M. Sun Q. Li X. Y. (2022). Attractive effects of previous form information on heading estimation from optic flow occur at perceptual stage. J. Vis. 22:18. doi: 10.1167/jov.22.12.18, PMID: 36413358 PMC9707032

[ref58] Wattam-Bell J. Birtles D. Nyström P. von Hofsten C. Rosander K. Anker S.. (2010). Reorganization of global form and motion processing during human visual development. Curr. Biol. 20, 411–415. doi: 10.1016/j.cub.2009.12.020, PMID: 20171101

[ref59] Xu L. H. Sun Q. Zhang B. Li X. (2022). Attractive serial dependence in heading perception from optic flow occurs at the perceptual and postperceptual stages. J. Vis. 22, 11–25. doi: 10.1167/jov.22.12.11, PMID: 36350629 PMC9652722

[ref60] Zhang H. Luo H. (2023). Feature-specific reactivations of past information shift current neural encoding thereby mediating serial bias behaviors. PLoS Biol. 21:e3002056. doi: 10.1371/journal.pbio.3002056, PMID: 36961821 PMC10075471

